# Exon expression profiling reveals stimulus-mediated exon use in neural cells

**DOI:** 10.1186/gb-2007-8-8-r159

**Published:** 2007-08-02

**Authors:** Adrienne E McKee, Nicola Neretti, Luis E Carvalho, Clifford A Meyer, Edward A Fox, Alexander S Brodsky, Pamela A Silver

**Affiliations:** 1 Department of Systems Biology, 200 Longwood Avenue, Harvard Medical School, Boston, Massachusetts 02115, USA.; 2 Department of Physics, Institute for Brain and Neural Systems, 182 Hope Street, Brown University, Providence, Rhode Island 02912, USA.; 3 Center for Computational Molecular Biology, 115 Waterman Street, Brown University, Providence, Rhode Island 02912, USA.; 4 Department of Applied Mathematics, 182 George Street, Brown University, Rhode Island 02912, USA.; 5 Department of Biostatistics and Computational Biology, Dana-Farber Cancer Institute, 44 Binney Street, Boston, Massachusetts 02115, USA.; 6 Department of Biostatistics, Harvard School of Public Health, 655 Huntington Avenue, Boston, Massachusetts 02115, USA.; 7 Department of Medicine, 200 Longwood Avenue, Harvard Medical School, Boston, Massachusetts 02115, USA.; 8 Department of Medical Oncology, Dana-Farber Cancer Institute, 44 Binney Street, Boston, Massachusetts 02115, USA.; 9 Department of Molecular Biology, Cell Biology, and Biochemistry, 185 Meeting Street, Brown University, Providence, Rhode Island 02903, USA.; 10 Center for Genomics & Proteomics, Center for Computational Molecular Biology, 70 Ship Street, Brown University, Providence, Rhode Island 02903, USA.

## Abstract

**Background:**

Neuronal cells respond to changes in intracellular calcium ([Ca^2+^]_i_) by affecting both the abundance and architecture of specific mRNAs. Although calcium-induced transcription and transcript variation have both been recognized as important sources of gene regulation, the interplay between these two phenomena has not been evaluated on a genome-wide scale.

**Results:**

Here, we show that exon-centric microarrays can be used to resolve the [Ca^2+^]_i_-modulated gene expression response into transcript-level and exon-level regulation. Global assessments of affected transcripts reveal modulation within distinct functional gene categories. We find that transcripts containing calcium-modulated exons exhibit enrichment for calcium ion binding, calmodulin binding, plasma membrane associated, and metabolic proteins. Additionally, we uncover instances of regulated exon use in potassium channels, neuroendocrine secretory proteins and metabolic enzymes, and demonstrate that regulated changes in exon expression give rise to distinct transcript variants.

**Conclusion:**

Our findings connect extracellular stimuli to specific exon behavior, and suggest that changes in transcript and exon abundance are reflective of a coordinated gene expression response to elevated [Ca^2+^]_i_. The technology we describe here lends itself readily to the resolution of stimulus-induced gene expression at both the transcript and exon levels.

## Abstract 2

Exon centric microarrays were used to resolve the calcium-modulated gene expression response into transcript-level an exon-level regulation.

## Background

The human genome is composed of 20,000-25,000 genes, which is roughly the same number as that of the mustard plant *Arabidopsis thaliana*, and only three to four times the number found in budding yeast [1-3]. These protein-coding segments are charged with the responsibility of establishing diverse organ types, such as brain, and with directing complex organ function within the context of stringent temporal and spatial constraints. As such, the human genome is faced with the task of encoding enormous complexity without the expected commensurate increase in gene number, begging the question of how such diversity and complexity is achieved.

Two prime strategies toward increasing complexity involve varying the abundance and the sequence structure of expressed mRNAs. Transcription and mRNA turnover affect the amount of whole transcripts present in a population. However, other forms of regulation affect the architecture of mRNA transcripts and significantly contribute to the complexity of gene expression. Alternative splicing and the processes of alternative transcription initiation and termination modify the nucleotide sequence of the transcript and therefore have the potential to influence both protein function and transcript stability [4-9].

The mammalian nervous system serves as a constructive model in which to study complex gene expression, because its anatomically and physiologically diverse cell types place unique demands on RNA processing. Recent *in situ *hybridization studies of expression in mammals have found that approximately 80% of all genes are expressed somewhere in the brain [10]. The brain also demonstrates the greatest amount of alternative splicing relative to other tissues [5,9]. Furthermore, the phenomenon of activity-dependent transcription, whereby cells react to transient changes in calcium (Ca^2+^) by affecting specific gene expression, is fundamental to the ability of the nervous system to institute long-term changes that affect learning and memory [11,12]. We are interested in how neural cells control and coordinate both of these demands - producing new transcripts and varying exon use - when they are challenged by differing environmental conditions.

In neural cells, Ca^2+ ^signaling has previously been shown to induce changes in the abundance of transcripts and in the diversity of specific mRNA isoforms [13-21]. Although Ca^2+^-induced transcription and transcript variation have both been recognized as important sources of neural gene regulation, the interplay between these two phenomena has not been evaluated on a genome-wide scale. Here, we use a high-density exon-centric microarray to examine gene expression in human IMR-32 neuroblastoma cells exposed to elevated intracellular Ca^2+ ^([Ca^2+^]_i_) concentrations. Both the influx of Ca^2+ ^through plasma membrane channels and the release of Ca^2+ ^from intracellular sources modulate neuronal gene expression [22,23]. We therefore analyzed cells treated over a time course of potassium chloride (KCl) membrane depolarization or treated with thapsigargin (TPG), a drug that inhibits endoplasmic reticulum (ER) Ca^2+ ^pumps, causing the release of ER-stored Ca^2+ ^ions into the cytoplasm [24].

We find that Ca^2+^-induced changes in exon abundance generate transcript variants of ion channels, neuroendocrine secretory proteins, and metabolic enzymes. Furthermore, we show that exons modulated by membrane depolarization are found in plasma membrane associated and Ca^2+ ^ion binding proteins. Our data link specific stimuli to expression behavior of distinct exons, and suggest that changes in transcript and exon abundance are reflective of a coordinated temporal transcriptional and post-transcriptional response to elevated [Ca^2+^]_i_.

## Results

### Exon-centric microarrays identify Ca^2+^-mediated changes in transcript and exon abundance

**Figure 1:**
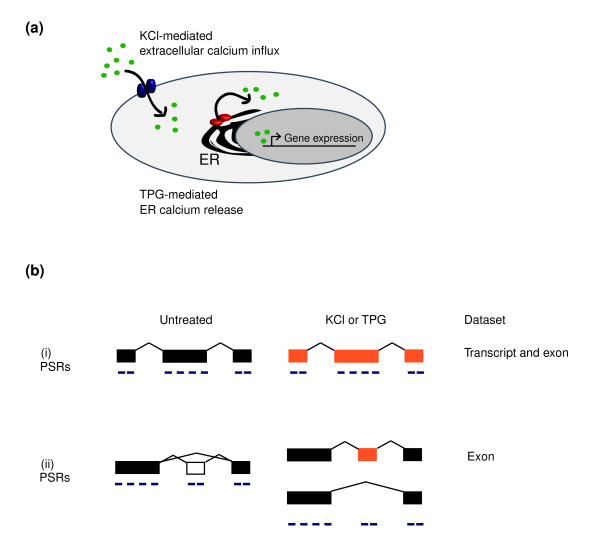
Exon-centric microarrays detect Ca^2+^-mediated changes in transcript and exon abundance. **(a) **Potassium chloride (KCl)-induced membrane depolarization and thapsigargin (TPG) treatment increase intracellular calcium (Ca^2+^) concentrations via extracellular Ca^2+ ^influx through plasma membrane channels and via release of Ca^2+ ^from intracellular stores, respectively. Ca^2+ ^(green) acts as a second messenger to effect gene expression. **(b) **Whole transcripts (i) and individual exons (ii) that exhibit a change in abundance in response to KCl or TPG can be identified because of the distribution of probes selection regions (PSRs; purple) into exonic regions (orange indicates a change in abundance). All of the PSRs of a transcript are used to determine transcript abundance, whereas the PSRs of an exon are used to determine exon abundance. The inclusion of an exon or transcript into one of two separate datasets (transcript or exon) was determined using a combination of fold change and statistical significance cut-offs (see text).

To examine the effects of Ca^2+ ^on a genome-wide scale, we treated human IMR-32 neuroblastoma cells with either depolarizing levels of KCl, which mediate an influx of extracellular Ca^2+ ^through plasma membrane channels, or with TPG, which causes intracellular Ca^2+ ^release from ER stores (Figure 1a). To gain additional information about the temporal gene expression response to elevated [Ca^2+^]_i_, we collected RNA from cells depolarized for a time series from 30 min to 24 hours. We chose the IMR-32 line [25] because several reports have demonstrated its usefulness for studying calcium dynamics, neuroblastoma differentiation, and cell stress and apoptosis [21,26-29]. We verified that our treatments were altering gene expression by examining transcripts that are known to be affected by these stimuli [21,30,31]. PCR performed on cDNA from untreated and treated cells demonstrated KCl and TPG induced transcription of the immediate-early *early growth-response factor 1 *(*EGR1*) gene, TPG induced transcription the *X-box binding protein *1 (*XBP1*) gene, and KCl and TPG induced splicing of the *plasma membrane calcium pump PMCA2*. We find that the expression of *EGR1 *is upregulated 14-fold upon 0.5 hours of KCl treatment but returns to basal levels by 6 hours after KCl addition. *EGR1 *is also upregulated upon prolonged TPG exposure. *XBP1 *expression, in contrast, is selectively upregulated 3.7-fold by TPG. See Additional data file 1 for quantification of transcript changes.

We used Affymetrix GeneChip Human Exon arrays [32] to investigate exon expression in unstimulated and stimulated IMR-32 cells. Figure 1b illustrates how the distribution of probe sets into exon regions enables evaluation of individual exons and whole transcripts. Biologic triplicate RNA samples were processed according to the Affymetrix whole genome amplification procedure (see Materials and methods, below) before hybridization to the exon array. All of the probes within a transcript were used to determine transcript abundance, whereas probe sets specific to an individual exon were used to determine exon abundance.

We generated two separate datasets (exon and transcript) from each triplicate set of arrays (Figure 1b). Differential expression between each time point and the initial, unstimulated time point was tested via a moderated *t*-test and an overall call for all times was generated via a moderated F-test [33]. *P *values from the F test were corrected for multiple testing to control the false discovery rate (FDR) [34]. To identify Ca^2+^-regulated RefSeq defined mRNAs, we applied two cut-offs: a ≥1.5 mean fold change and a FDR of 0.01 for the exon dataset and a FDR of 0.005 for the transcript dataset. Using these criteria, we find that 5,139 (KCl) and 8,533 (TPG) RefSeq transcripts contain one or more exons that change in abundance upon stimulation for at least one time point. Through separate analysis of all of the probes in a RefSeq transcript, we identified 1,505 (KCl) and 3,489 (TPG) whole transcripts that exhibited a change in abundance upon treatment. Full lists of significantly changing transcripts and exons are presented in Additional data files 2 and 3.

**Figure 2:**
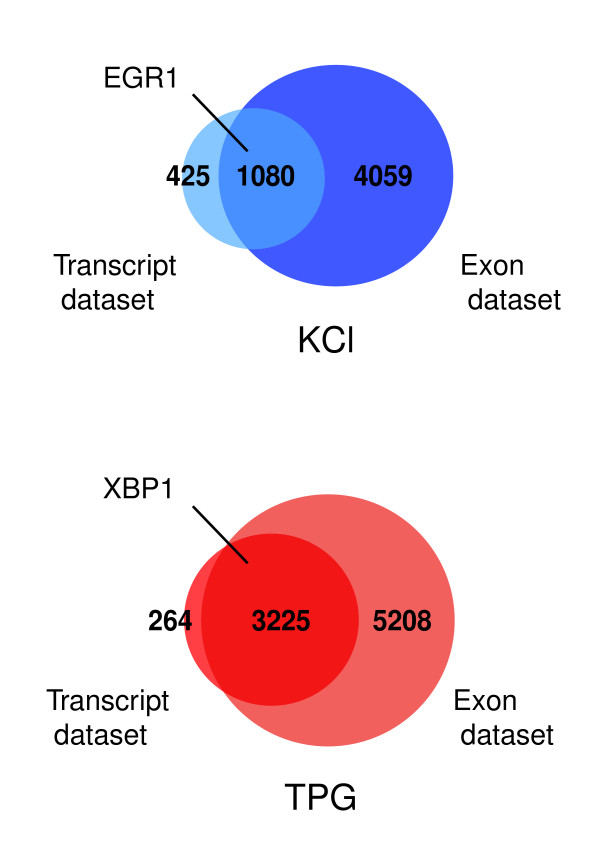
Thousands of genes are affected by elevated intracellular calcium. Venn diagrams show the intersection of potassium chloride (KCl) and thapsigargin (TPG) transcript and exon datasets. Indicated in bold are the number of transcripts in each dataset. All significantly changing transcripts identified in the KCl time course are represented. A subset of transcripts were scored as changing in abundance, even though individual exons within these transcripts were not scored as showing statistically significant changes in expression levels. This is a statistical artifact that results from using data from only a few probes per exon (typically four), but many more probes per transcript. See Materials and methods. Light blue indicates KCl whole transcript changes, and dark blue indicates transcripts that display a change in exon abundance upon KCl. Dark red indicates TPG whole transcript changes, and light red indicates transcripts that display a change in exon abundance upon TPG. Identification of early growth-response factor 1 (*EGR1*) and X-box binding protein 1 (*XBP1*) in specific datasets is indicated.

Venn diagrams showing the overlap of the exon and transcript datasets for KCl and TPG treatments are presented in Figure 2. All significantly changing transcripts identified throughout the KCl time course are represented in a single diagram. For each diagram, the intersection represents those transcripts that demonstrated an abundance change through both interrogation of their individual exons (exon dataset) and of all of the probes comprising that transcript (transcript dataset). Notably, *EGR1 *is found in the intersection of the KCl transcript and exon datasets, whereas *XBP1 *is found in the intersection of the TPG transcript and exon datasets, thereby verifying that the array and our analytical approach were capable of identifying transcripts that are known to be affected by these stimuli (Figure 2).

Because transcript estimates are a direct composite of individual exon use, we expected the transcript and exon datasets to overlap significantly. Indeed, 72% (KCl) and 91% (TPG) of the components of the transcript datasets were recognized as containing significantly changing exons (Figure 2). In addition to representing transcript abundance changes, this region of intersection is likely to signify those genes that are undergoing changes both in abundance and in transcript architecture. The outlying constituents of the exon dataset, in contrast, show an exon change without a commensurate change in transcript abundance, indicating that only a subset of exons in a transcript are affected. Finally, the outlying fraction of the transcript datasets represents those transcripts for which no single exon passed the selection criteria, presumably because of greater variability at the probe set level (see Materials and methods, below). Fewer exons were identified as changing in transcripts among the exon datasets than in the Transcript datasets, as shown in Additional data file 4.

### Ca^2+^-mediated changes in transcript abundance are associated with functional gene categories

**Figure 3:**
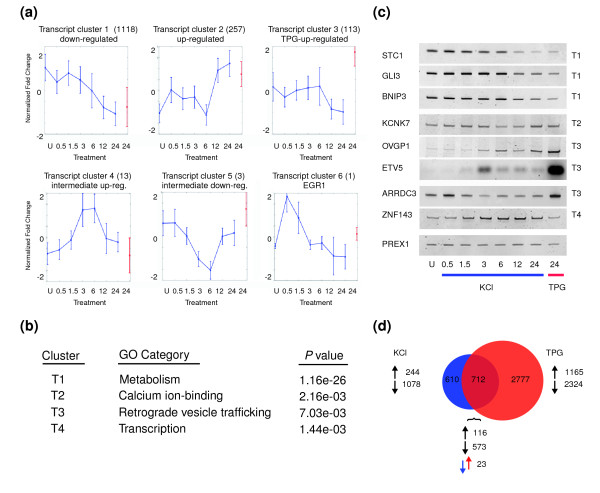
Calcium-mediated changes in transcript abundance are enriched for functional gene categories. **(a) **Hierarchical clustering of the potassium chloride (KCl) transcript dataset identifies six major patterns of gene expression over the 24-hour time course. The number of transcripts contained in each cluster is designated in parentheses. Shown in each plot is the averaged expression behavior of all transcripts in the indicated cluster, with error bars indicating the deviation from the mean for the entire cluster. Expression behavior of each transcript was obtained by subtracting the mean of all points at all times from each expression value in the original time series, and dividing by the standard deviation. KCl-treated time points are indicated in blue. Thapsigargin (TPG) treatment is indicated in red. **(b) **Gene Ontology (GO) analyses of the transcripts in each cluster reveals functional enrichment in clusters T1 to T4. **(c) **Polymerase chain reaction performed on cDNA from treated and untreated IMR-32 cells validates changes in transcript abundance predicted by the microarray data. *PREX1*, a gene whose abundance was not predicted to change, served as a normalization control. **(d) **Transcripts regulated by KCl stimulation at 24 hours are similarly affected by TPG treatment. Numbers indicate upregulated and downregulated transcripts. Among those transcripts affected by both KCl and TPG, 116 were upregulated and 573 were downregulated. *ARRDC3*, arrestin domain containing 3; *BNIP3*, BCL2 adenovirus E1B 19 kDa interacting protein 3; *ETV5*, ets variant gene 5; *GLI3*, GLI-Kruppel family member 3; *KCNK7*, potassium channel, subfamily K, member 7; *PREX1*, phosphatidylinositol 3,4,5-trisphosphate-dependent RAC exchanger 1; *OVGP1*, oviductal glycoprotein 1; *STC1*, stanniocalcin 1; *ZNF143*, zinc finger protein 143.

In order to characterize the transcripts that are regulated by KCl, we identified groups of transcripts that exhibit similar patterns of expression between stimuli and across time points. Using hierarchical clustering with a correlation analysis on the expression of constituents of the KCl transcript dataset [35], we identified specific clusters of genes that respond to KCl and TPG in similar manners (Figure 3a). Each plot in Figure 3a represents the averaged expression behavior of the transcripts in the indicated cluster. Error bars indicate the deviation from the mean for the entire cluster. The size of each cluster varies. For example, 13 genes were shown to be upregulated by KCl treatment at the intermediate time points of 3 and 6 hours, with little to no change at early and late time points (Figure 3a, cluster T4). The sole transcript in cluster T6 is the immediate-early response gene *EGR1 *(Figure 3a, cluster T6).

Gene ontology (GO) [36,37] analyses performed on the set of genes in each cluster show that the four largest clusters (clusters T1 to T4; Figure 3a) are enriched for different functional gene classes, indicating that functionally related groups of genes are co-regulated upon KCl and TPG stimulation. Representative enriched classes for each cluster are listed in Figure 3b. Significant GO categories for each class are presented in Additional data file 5.

mRNAs encoding proteins that are involved in cellular metabolism, ion transport, retrograde vesicle-mediated transport from the Golgi to the ER, and transcription are over-represented among transcripts affected by elevated [Ca^2+^]_i_. These gene classes exhibit different expression profiles over the KCl time course. Metabolism genes, including lipid, carbohydrate, and nucleic acid synthesizing genes, are downregulated upon increased KCl exposure, perhaps reflecting an overall decrease in energy consumption with increased exposure to cellular stress. In contrast, transcripts that encode proteins at the plasma membrane and ion transporters, including Ca^2+ ^ion binding proteins, are upregulated, pointing to an augmented requirement for ion homeostatic regulating factors with prolonged exposure to elevated [Ca^2+^]_i_. Transcripts displaying increased expression under ER stress conditions (cluster T3; Figure 3a) are enriched for genes encoding proteins that are involved in retrograde vesicle-mediated transport from the Golgi to the ER, indicating a greater demand for proteins involved in the secretory pathway in response to ER stress. Finally, transcription factors are over-represented among genes that are transiently upregulated during intermediate KCl time points (cluster T4; Figure 3a). Genes in cluster T4 include the transcription factors GA binding protein, beta subunit 2 (*GABPB2*), zinc finger protein 143 (*ZNF143*), and peroxisome proliferator-activated receptor gamma (*PPARG*). These transcription factors are of particular interest because the timing of their expression suggests that they are secondary effectors of calcium signaling. Notably, *PPARG *and the GABPB2-interacting protein *GABPA *were recently reported to be regulated in an activity-dependent manner in primary rat neurons [38].

We validated transcript abundance changes predicted from the microarray data by quantitative PCR (qPCR). Overall, we found very good accordance between PCR and microarray data using the criteria selected (see Additional data file 6 [part B] for a receiver operating characteristic plot of this data). Seventeen out of 21 transcripts that passed both the fold and *P *value criteria demonstrated a change by qPCR, whereas nine out of nine that were not predicted to show a difference in abundance did not. Examples of PCR-verified changes in transcript abundance, highlighting downregulated, upregulated, and differentially regulated transcripts from transcript clusters 1 to 4 are shown in Figure 3c. Quantification of data shown in Figure 3c is presented in Additional data file 7.

We validated previously unreported instances of Ca^2+^-modulated transcript changes, such as decreased Ca^2+^-mediated expression observed for stanniocalcin 1 and increased expression of oviductal glycoprotein 1. We also verified examples of Ca^2+^-modulated transcripts that have been detected in rat cerebellar neurons [39,40] and in human SH-S5Y5 neuroblastoma cells in response to ER stress [31]. Our data provide new information about temporal expression behavior that is only distinguished through detailed time course analyses.

Thapsigargin treatment causes Ca^2+ ^influx into the cytoplasm by a mechanism that is distinct from KCl, and was therefore used as a point of comparison. TPG affected a greater number of transcripts than did KCl membrane depolarization (Figure 2).

However, more than half of the transcripts affected by KCl were similarly regulated by TPG exposure (Figure 3d). This finding points to a common response mode to different forms of [Ca^2+^]_i_, which is primarily transcriptional repression rather than transcriptional stimulation. A subset of mRNAs exhibits opposing behavior under the two stimuli, and may represent responses that are unrelated to Ca^2+^. For example, transcripts of arrestin-domain-containing-3 are downregulated under KCl but upregulated by TPG (Figure 3c). The principal feature of genes uniquely affected by TPG is their functional association with the secretory system.

### Exon-centric microarray data provide an entry-level screen of Ca^2+^-mediated changes in splice form diversity

To explore instances of Ca^2+^-mediated exon-specific regulation, we examined the KCl and TPG exon datasets. Strikingly, many more transcripts demonstrated changes in expression levels of only a subset of exons, as compared with those showing changes throughout the entire transcript, suggesting that a pronounced alteration in exon usage occurs in response to KCl and TPG. Fully 4,059 transcripts contain exons that show a change in abundance upon KCl stimulation without a commensurate change in overall transcript abundance (Figure 2).

We hypothesized that an increase in the abundance of either single or a few exons without concomitant increase in the exons comprising the rest of the transcript might arise from several sources. Exons from these transcripts may represent alternative splicing events or may represent alternative transcription initiation or termination. Changes in exon abundance may also reflect a change in transcript abundance that is not detected in the case that probe sets throughout the transcript are of poor quality or exhibit cross-hybridization. Alternatively, the identified probesets in the exon dataset may result from other 'noise' in the data acquisition or analysis. To verify that microarray-predicted changes in exon expression were indicative of true exon modulation, we analyzed a sampling of 23 exons by quantitative PCR, using cDNA from independently prepared RNA samples. We chose exons that reflected different types of expression behavior (upreguilated or downregulated, as estimated by the array data). Some exons failed both the *P *value and fold change criteria, some passed one or the other, and others passed both criteria. As shown in Additional data file 6 (parts C and D), we found good accordance between qPCR and microarray data using the criteria selected. Twelve of 16 exons that passed both the 1.5-fold and 0.01 *P *value criteria demonstrated a change by qPCR, whereas six out of seven that were not predicted to show a difference in abundance did not.

We further validated instances of regulated exon use by testing for alternative splicing in the vicinity of affected exons. We hypothesized that a change in the abundance of a specific exon would indicate that one or more splice variants varied the incorporation of the exon. To detect instances of alternative splicing, we performed PCR on cDNA from KCl and TPG treated cells using primer sets that spanned the exon(s) of interest. We targeted those exons that differed upon stimulation without a predicted change in transcript abundance (Figure 2, unique to exon dataset). We focused on internal exons, as opposed to exons located at the end or beginning of a transcript, that exhibited a consistent pattern of behavior over multiple time points or between KCl and TPG treatments. Despite not knowing the overall architecture of the isoforms targeted, we found that 11 out of 19 reactions yielded multiple transcripts. Of these 11, seven showed a trend in exon incorporation over the KCl time course or between treatments, reflecting regulated exon use in the pattern predicted by the array. To establish the rate of false negatives in our system, we similarly examined 20 exons that were excluded by both cut-offs. Only two reactions exhibited multiple splice forms, both of which varied over time (data not shown), indicating a low false-negative rate.

### Ca^2+^-induced changes in exon abundance generate transcript variants of ion channels, neuroendocrine secretory proteins, and metabolic enzymes

**Figure 4:**
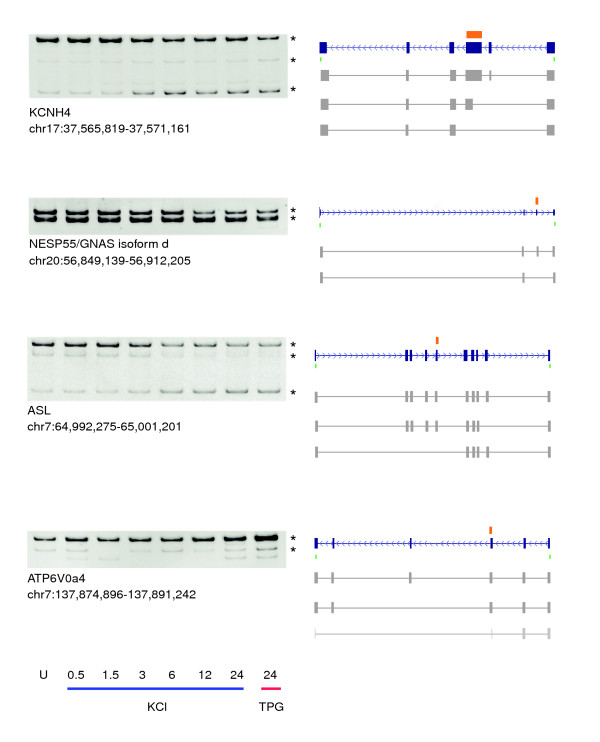
PCR validation of predicted changes in exon abundance identifies calcium-mediated splice form diversity. Microarray data detected decreases in the expression of specific exons (indicated in orange) within *KCNH4*, *NESP55*, *ASL*, and *ATP6V*_0_*a4*. For each gene, polymerase chain reaction was performed on cDNA from potassium chloride (KCl) or thapsigargin (TPG) treated cells using primers (designated by green bars) to amplify across the exon of interest. Multiple transcript variants were detected, indicating alternative splicing in the vicinity of the identified exons. Images depicting RefSeq transcript architecture were obtained through the UCSC Genome Browser [66,67]. Sequenced isoforms are indicated (*) and are represented by dark gray boxes. For ease in alignment, intron sequences are shown. The predicted sequence of the smallest *ATP6V*_0_*a4 *variant is shown in light gray. Isoform quantification is presented in Additional data file 9. *ASL*, argininosuccinate lyase; *ATP6V*_0_*a4*, ATPase, H^+ ^transporting, lysosomal V0 subunit a4; *KCNH4*, potassium voltage-gated channel, subfamily H (eag-related), member 4; *NESP55*, neuroendocrine secretory protein 55.

Figure 4 shows examples of Ca^2+^-mediated changes in transcript structure resulting from alternative splicing of identified exons. Our array data detected a Ca^2+^-mediated decrease in abundance of certain internal exons from the transcripts encoding potassium voltage-gated channel, subfamily H, member 4 (*KCNH4*), the neuroendocrine secretory protein 55 (*NESP55*), the ATPase, H^+ ^transporting lysosomal V_0 _subunit a4 (*ATP6V*_0_*a4*), and the argininosuccinate lyase (*ASL*). In these cases of decreased abundance, we expected to detect shorter transcript variants (lacking part or all of the identified exons). We amplified the predicted full-length amplicon, but we also observed additional transcript variants. Indicated splice forms were subcloned and sequenced to verify their identities (Figure 4). Although alternative splicing of the specific exons identified in *NESP55*, *ATP6V*_0_*a4*, and *ASL *have been detected in expressed sequence tags, to our knowledge, regulation of exon variation upon a stimulus change has not been reported for these genes. The alternative use of the exon identified in *KCNH4 *represents a new finding of both Ca^2+^-induced regulation and of alternative splicing.

The alternative splicing events seen in *KCNH4*, *ATP6V*_0_*a4*, and *ASL *have effects within the protein-coding sequence of these transcripts. The *KCNH4 *potassium channel is a member of the voltage-gated potassium H subfamily, members of which have been shown to control the resting potential of membranes [41]. The smallest transcript variant lacks both the exon identified by the array and the upstream proximal exon (Figure 4). The intermediate splice form represents partial use of the exon indicated by the array data. In the absence of other upstream changes, exon exclusion would result in a premature stop codon (PTC). Both of these *KCNH4 *isoforms therefore may be candidates for non-sense mediated decay (NMD), because the ensuing PTCs are more than 55 nucleotides from the downstream exon-junction complex [42]. Should these transcripts evade NMD, we predict that the Ca^2+^-induced alternative splicing event would generate a truncated protein that still contains the membrane-spanning segment but that lacks much of a large cytoplasmic region responsible for intracellular protein-protein interactions. Protein interactions on the cytoplasmic face of the channel regulate its assembly, gating, and conduction properties [43]. Therefore, molecular alteration of the carboxyl-terminal region is likely to affect protein function [44] and may influence the re-establishment of ion homeostasis.

A truncated form of *ASL *may similarly affect protein-protein interactions of this functional homotetramer [45]. In neural cells, *ASL *catalyzes the cleavage of argininosuccinate to produce L-arginine, thereby regulating the amount of free arginine available for nitric oxide production [46]. Nitric oxide is a critical second messenger in the nervous system, with both neuroprotective and neurodegenerative roles [47,48]. Regulated splicing of *ASL *may therefore influence nitric oxide through modulation of L-arginine production. Several splice forms of *ASL *appear to be regulated in a stimulus-dependent manner. We identified a transcript variant that lacks the indicated exon (smallest *ASL *isoform; Figure 4). This transcript is also missing three additional upstream exons, but because the PTC occurs proximal to the downstream exon-junction it probably evades NMD. Also identified in our PCR analysis is an intermediately sized isoform (Figure 4) that retains the protein-coding frame.

The *ATP6V*_0_*a4 *gene encodes a component of the V-type H^+ ^ATPase that is involved in acidification of intracellular compartments and in the recycling of neurotransmitters [49,50]. The V_0_a4 gene is one of at least four distinct loci that encode the membrane spanning H^+ ^channel of the V_0 _complex. Although different V_0_a loci are thought to be expressed in tissue-specific manners [50], our data point to a role for alternative splicing in generating subunit diversity. We uncovered three variants of ATP6V_0_a4 upon early and late KCl treatment, two of which are present throughout most of the time course (Figure 4). Manual inspection of expressed sequence tag and gene prediction databases for *ATP6V*_0_*a4 *isoforms in the region amplified uncovered several transcript variants, some of which exclude a portion of the identified exon, thereby generating shortened, but in-frame transcripts. Sequencing revealed that the shorter form indicated in Figure 4 maintains the protein-coding sequence. Stimulus-induced splicing of *ATP6V*_0_*a4 *could modify the protein to alter its distribution, or membrane interactions, influencing proton cycling.

In contrast, the alternative splicing event in *NESP55 *(also known as G-protein-stimulating alpha isoform d) affects the 3'-untranslated region of the *NESP55 *transcript. The secreted NESP55 glycoprotein is entirely encoded in its 5' exon, but shares exons of its 3'-untranslated region with protein-coding exons of other *GNAS *transcripts [51-53]. We found that the abundance of *NESP55 *exon 3 decreases in later KCl time points and upon prolonged exposure to TPG (Figure 4). Sequence data confirm that the smaller isoform lacks exon 3 (Figure 4). Exon modulation within the untranslated region may suggest a role for stimulus-regulated mRNA stability.

Eleven out of 19 transcripts containing exons predicted to change over the time course or upon TPG treatment demonstrated multiple transcript variants by PCR. Array data distinguished a Ca^2+^-mediated decrease in abundance of internal exons from the transcripts encoding syntaxin-binding protein 1 (*STXBP1*) and CDK5 regulatory subunit associated protein 3 (*CDK5RAP3*), as well as a stimulus-mediated increase in an internal exon of the nitric oxide synthase 2A gene (*NOS2A*). When examined by PCR using primer sets that amplified across the identified exon, exons of *STXBP1 *and *CDK5RAP3 *demonstrated increased exclusion, whereas the exon of *NOS2A *showed increased inclusion. These alternative splicing findings are in agreement with the nature of the behavior of the exon, as evaluated by the array. Shown in Additional data file 9 are PCR results and splice form quantification of seven exons tested that showed a trend in incorporation over the KCl time course or between treatments. Also presented in Additional data file 9 are PCR results for four other transcripts that exhibited multiple splice forms in the vicinity of the identified exon but did not demonstrate a clear pattern of regulated exon use that could be attributed to the exon in question.

### Exons modulated by membrane depolarization are found in plasma membrane-associated and Ca^2+ ^ion-binding proteins

**Figure 5:**
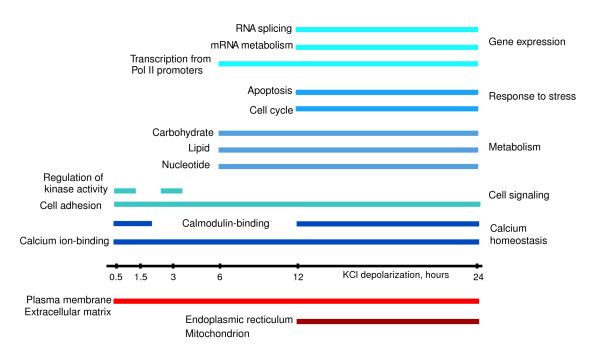
Transcripts affected by calcium-mediated changes in exon abundance show enrichment for cellular component and functional gene classes. Gene Ontology categories over-represented within the exon dataset at each potassium chloride (KCl) time point are shown. Genes encoding calcium ion binding proteins and those proteins found at the plasma membrane or as a part of the extracellular matrix are affected throughout the time course of KCl depolarization. In contrast, other classes of genes are represented at either early or late time points. Descriptions of similar gene classes are listed. The number of transcripts affected by predicted changes in exon abundance, along with associated *P *values for each gene class, are listed by time point in Additional data file 8.

In order to investigate the nature of the exon abundance changes on a larger scale, GO analyses were performed on the exon dataset (Figure 5). We find that transcripts harboring KCl-mediated exon abundance changes are enriched for plasma membrane-associated and Ca^2+ ^ion binding proteins. Indeed, we find enrichment within the exon dataset for the same gene classes as found for the transcript dataset, such as metabolism, Ca^2+ ^ion binding, and transcription. Because the exon dataset mainly encompasses the transcript dataset, these results are expected. However, whereas GO enrichments were seen in the later KCl time points within the transcript dataset, Ca^2+ ^ion binding genes are over-represented throughout the early, intermediate, and late KCl time points within the exon dataset. Notably, we also uncover instances of calmodulin-binding proteins in the exon dataset that were not identified through our transcript analysis. Together, these ontologic findings suggest that the Ca^2+^-mediated gene expression response extends on a genome-scale to affect exons of different Ca^2+^-associated genes.

We additionally observe enrichment for functional gene classes. Reflective of a change in signaling programs upon KCl addition, early time points (0.5 and 3 hours) are over-represented by genes that encode kinases and kinase-regulating proteins (Figure 5). As exposure to elevated [Ca^2+^]_i _continues, changes in expression affect genes encoding proteins that are involved in metabolism, cell cycle control, and apoptosis. Enrichment of genes that are involved in lipid, carbohydrate, and nucleic acid synthesizing genes, as well as cyclins and cyclin-dependent kinases and proapoptotic genes, are downregulated or contain exons that are downregulated upon KCl exposure. Genes with known roles in regulating gene expression through transcription, mRNA splicing, or RNA processing are also among factors that are altered at later time points. These data point to extensive stimulus-driven modulation of exon use, beyond that describing transcriptional changes.

### Exons at transcript termini and those displaying alternative splice site choice are enriched in the exon datasets

The KCl and TPG Exon datasets are biased for terminal exons compared with their incidence in RefSeq transcripts. To obtain an idea of how different portions of transcript structure are subject to regulation, we annotated all RefSeq exons as initial, internal, or final according to their presence in RefSeq transcripts. We then scored the constituents of the exon datasets by these assignments. Table 1 shows that exons in the KCl and TPG datasets are enriched at transcript termini positions.

**Table 1:** Distribution of exon positions in RefSeq transcripts and in the exon datasets

Exon position	Percentage in RefSeq database	Percent in KCl exon	*P *value	Percentage in TPG exon	*P *value
Initial	9.96	12.79	3.550 × e^-15^*	12.63	5.030 × e^-11^*
Internal	79.79	67.24	1	68.98	1
Final	10.25	19.97	0*	18.40	0*

RefSeq exons were classified as initial, internal, or final according their presence in RefSeq transcripts. Constituents of the potassium chloride (KCl) and thapsigargin (TPG) exon datasets were mapped to these definitions. *P values showing statistical significance for enrichment of transcript features through a binomial test.

We next asked whether different exon types are enriched in our datasets, as compared with their incidence in RefSeq transcripts. We further annotated all RefSeq exons as either constitutive or alternative according to their presence in RefSeq transcripts. We subdivided alternative exons into cassette, or alternative 5' or 3' splice site choice categories and then scored the constituents of the exon datasets by these assignments. At all transcript positions, the KCl and TPG exon datasets are biased for exons displaying alternative 5' or 3' splice site choice (Table 2). This increase in distribution toward alternative splice site choice exons was accompanied by a decrease in constitutive and alternative cassette exons (data not shown). These significant biases were also observed when we compared KCl and TPG exons with the set of GenBank mRNAs (data not shown). Our findings indicate that exons exhibiting alternative splice site choice are subject to greater degrees of change than are other exon forms. Overall, these observations suggest that the selected exons are subject to Ca^2+^-mediated regulation of post-transcriptional processes, including alternative splicing and 3' processing.

**Table 2:** Distribution of exons exhibiting alternative splice site choice in RefSeq transcripts and in the Exon datasets

Exon position	Type of alternative splice choice	Percentage in RefSeq database	Percentage in KCl exon	*P *value	Percentage in TPG exon	*P *value
Initial	5'	24.32	28.14	1.367 × e^-03^*	27.70	8.081 × e^-05^*
Initial	Alternative start	25.25	27.18	6.386 × e^-02^*	30.07	6.674 × e^-08^*
Internal	5'	5.31	8.02	1.179 × e^-13^*	6.53	4.935 × e^-11^*
Internal	3'	5.32	8.05	8.393 × e^-14^*	6.77	9.659 × e^-15^*
Final	Alternative termination	23.43	25.63	1.574 × e^-02^*	28.55	1.110 × e^-16^*
Final	3'	22.92	23.27	3.515 × e^-01^	24.83	7.685 × e^-04^*

RefSeq exons were classified as exhibiting 5' or 3' alternative splice site choice according their presence in RefSeq transcripts. Constituents of the potassium chloride (KCl) and thapsigargin (TPG) exon datasets were mapped to these definitions. *P values showing statistical significance for enrichment of alternative splice site choice exons at initial, internal, and final positions through a binomial test.

## Discussion

The generation of molecular complexity achieved by cells of higher eukaryotes relies on transcriptional and post-transcriptional processes. The capacity of cells to modify multiple forms of gene regulation upon changing environmental conditions allows the formation of situation-specific transcriptomes. Previous work from researchers studying different neuronal systems has identified Ca^2+^-regulated transcriptional programs as well as instances of Ca^2+^-regulated transcript variation due to modified exon use [8,11,19,54]. Prior to recent advances in microarray technology, however, these related forms of gene regulation were characterized independently on a single gene basis. As a first step toward elucidating Ca^2+^-regulated transcription and transcript variation in the same study, we used high-density, whole-genome exon arrays to profile the Ca^2+^-responsive exon complement of stimulated IMR-32 neuroblastoma cells. In this work, we present an analysis of transcript and exon expression affected by KCl membrane depolarization or TPG.

### Changes in exon expression report on multiple forms of gene regulation

Exon level expression estimates encompass changes in whole transcript abundance but they additionally provide a measure of exon abundance that can be evaluated independently of transcript structure. Our analysis reveals that thousands of genes are affected by elevated [Ca^2+^]_i _over the time course and stimuli addressed (Figure 2). Many of these genes are regulated at the whole transcript level, but the majority undergoes modulation of only a subset of exons (Figure 2).

It is important to note that the inclusion of an exon in our dataset may reflect an abundance change in the entire transcript, alteration in exon use through alternative splicing, alternative transcriptional initiation or termination, or a combination of these events. In this regard we consider identified exons as primary candidates for further investigation by PCR. However, when we account for transcript expression, we are able to identify numerous alternative splicing events from exon-level expression data (Figure 4).

In this work, we specifically targeted exons of transcripts that did not demonstrate an abundance change in order to reduce the likelihood of confounding transcriptional differences. This strategy is similar in principle to the splice index approach utilized by Gardina and coworkers [32] and Clark and colleagues [55] but it requires a constant level of transcription. We identified regulated alternative splicing events in 37% of cases tested (7/19) and multiple splice isoforms were present in 58% (11/19) of cases tested. Our findings indicate that elaborate filtering is not necessary to obtain a validation rate comparable to that of the splicing index strategy when using the same array platform [32]. More recent exon-centric array analyses of tissue-specific exon expression have considerably improved validation [55], although whether this is due to use of mismatch probe sets or to the higher prevalence of splice form differences between disparate tissues has not been evaluated.

Our data suggest that stimulus-induced changes in alternative splicing act as a major contributor to gene regulation. The capacity to resolve an exon-level change onto transcript structure, however, remains a challenge; our PCR validation strategy failed to uncover the nature of splicing isoforms in 63% (12/19) of cases tested. Notably, this parallels the false-positive rate previously reported for exon-centric arrays [32]. When alternative splicing is more complex than a single exon inclusion or exclusion event or when the transcript is targeted for decay, PCR performed on adjacent exons may not amplify multiple products depending on the location of the primer sets. Indeed, because exon expression measures the total transcriptional output of an exon, complicated splicing patterns may be difficult to unravel. This issue is overcome by exon-junction platforms, in which the splice forms in question are directly interrogated [6]. The exon-junction format has typically been more successful in discerning tissue-specific [4,5,9] or condition-specific splicing [56] than the exon-centric platform. However, because exon-junction arrays require *a priori *knowledge of transcript architecture, a trade-off must be made in the discovery of novel transcript variants for the measurement of known splicing events. In the future, combining detailed understanding of transcript architecture with each individual exon's behavior will probably improve the detection rate of splicing events from exon-centric array data.

Analyses of exons by transcript position indicate that more than 30% of changes occur at transcript termini (Table 1). Our data also demonstrate a minor yet significant bias for alternative splice site exon use at all transcript positions (Table 2). These finding have two implications. First, they indicate that the modification of alternative splice site choice is subject to dynamic regulation. Conditions of elevated [Ca^2+^]_i _may affect the phosphorylation status of splicing factors to utilize alternate or cryptic splice sites [57]. Second, they suggest that regulated alternative transcriptional initiation and 3' end processing have impacts on large numbers of genes. Recent investigations into promoter elements have found that more than 40% of human genes have alternative promoters [58,59]. An equal number of human genes is also subject to alternative polyadenylation [60], indicating that these two forms of gene regulation are widely utilized. Although tissue-specific factors are certainly critical in determining initiation and polyadenylation sites, situation-specific regulation may also play a considerable role in generating transcript diversity.

### KCl membrane depolarization directs gene expression of Ca^2+ ^ion binding proteins

Importantly, our finding that exons affected by KCl and TPG occur in physiologically meaningful transcripts demonstrates that a coordinated gene expression response, involving both transcription and splicing, occurs when neural cells are challenged by elevated [Ca^2+^]_i _conditions. GO analyses performed on the KCl transcript and exon datasets revealed functional enrichment for proteins that are involved in the cellular response to membrane depolarization. Although GO results from the exon dataset highlight the same categories as in the transcript dataset, they additionally register gene classes that are either absent from the transcript dataset or are found throughout the time points examined. At the foremost, genes encoding Ca^2+ ^ion binding proteins are enriched throughout the time course of KCl stimulation (Figure 5). Because membrane depolarization induces Ca^2+ ^influx, elevated [Ca^2+^]_i _increases the cellular requirement for proteins that function to restore ion balance, in the form of channels and pumps or as buffering agents. Our combined data suggest that transcriptional changes alone, as measured using all of the probe sets of a transcript, account for only a portion of the total gene expression response. Forms of exon modulation, including alternative splicing, are additionally responsible for important regulation of gene expression.

### Exon expression profiling connects cellular stimuli to specific exon behavior

We identified and validated instances of regulated alternative splicing in genes that may have an impact on neural cell function or physiology. *KCNH4 *transcripts undergo exclusion of an exon affecting the carboxyl-terminal portion of the protein (Figure 4). *In vitro*, members of the potassium voltage-gated channel H subfamily have been shown to control the resting potential of membranes [41] by pumping potassium out of the cell. Because intracellular protein-protein interactions regulate channel properties, splicing in this region may modify the function of the channel, thereby affecting ion homeostasis. Regulated splicing of the membrane-spanning component of the V_0 _proton pump may also have an impact on ion balance, because both proton concentration difference and membrane voltage are generated by the V-type H^+ ^ATPase [50]. Conditions of elevated [Ca^2+^]_i _may also affect L-arginine metabolism through alternative splicing of *ASL*. Collectively, these data provide genome-scale evidence that alternative splicing is utilized to respond to cellular stimuli.

### KCl membrane depolarization and TPG show similar yet distinct effects on gene expression

KCl and TPG treatments affect both overlapping and specific gene sets in IMR-32 neuroblastoma cells. Genes that modulate Ca^2+ ^ion balance are upregulated by both conditions (Figure 3b). The two stimuli also lead to a decrease in the transcriptional abundance of metabolism-related and proapoptotic mRNAs (Figures 3b and 5, and data not shown). TPG treatment appears to enhance the expression of genes that are also affected by KCl. We additionally find that alternative splicing is similarly regulated by the two Ca^2+ ^stimuli (Figure 4), at least for the cohort of genes confirmed by PCR. These findings indicate the presence of gene regulatory factors that are responsive to elevated [Ca^2+^]_i _and not necessarily to the mode of ion imbalance. It is important to note as well that neuroblastoma cells subjected to prolonged increased Ca^2+ ^ion balance may be activating cell death gene expression responses, thereby showing secondary effects of elevated calcium signaling [31,61]. Although not the specific focus of this study, these data may serve to identify candidate exons that are involved in the cell death activation pathways.

A large subset of mRNAs shows TPG-specific modulation (Figure 3d). TPG treatment specifically upregulates the transcriptional profile of genes involved in ER to Golgi transport and secretory pathway (Figure 3b). This is likely indicative of activation of the unfolded protein response previously shown to be induced by ER damage in neuroblastoma [31,62]. Because the ER is involved in long-term calcium signaling events and because ER-calcium misregulation is a key factor in neurodegeneneration [23], the identification of transcripts associated with ER damage is critical to our understanding of disease pathologies. Thus, these data may also serve to identify exons that are regulated by alternative splicing or alternative transcriptional events that are specifically involved in the ER stress response.

## Conclusion

Gene expression is a composite of the output of transcription and of additional processes that modulate the exon composition of individual mRNAs. Although differences in transcript abundance have routinely been used as indicators of cell activity, it is clear that both the amount and the sequence diversity of transcripts are important contributors to the expression complexity of human cells. Strategies examining multiple forms of gene expression changes are likely to provide a broader definition of cell behavior than are studies aimed at singular expression responses. These data expand our understanding of how cells respond to elevated [Ca^2+^]_i _at the transcript and individual exon levels.

## Materials and methods

### Cell culture, KCl membrane depolarization, and TPG treatment

Human neuroblastoma IMR-32 cells (ATCC, Manassas, VA, USA) were cultured at 37°C in a humidified atmosphere with 5% carbon dioxide in Eagle's minimum essential media (EMEM; ATCC), modified to contain the following: Earle's balanced salt solution, nonessential amino acids, 2 mmol/l L-glutamine, 1 mmol/l sodium pyruvate, and 1.5 g/l sodium bicarbonate, with 10% fetal bovine serum (Invitrogen, Carlsbad, CA, USA) and penicillin/streptomycin. Before membrane depolarization or ER stress studies, cells were grown in EMEM serum to 70% to 90% confluency and were then serum starved for 48 hours with two changes of serum-free EMEM. Stimulus treatments were performed as described previously [21], except that a HEPES-KCl depolarization solution was prepared using tissue culture grade reagents (Sigma, St. Louis, MO, USA). IMR-32 cells were treated with 50 mmol/l KCl for 0.5, 1.5, 3, 6, 12, or 24 hours, or were exposed to 5 μmol/l TPG (Tocris Bioscience, Ellisville, MO, USA) for 24 hours.

## RNA extraction and reverse transcription PCR

Total cellular RNA was extracted using TRIzol Reagent (Invitrogen), in accordance with the manufacturer's instructions. RNA was further purified using 2 U/ml Turbo DNase (Ambion, Austin, TX, USA) and then applied to and eluted from RNeasy Mini Spin Columns (Qiagen, Valencia, CA, USA). cDNA synthesis from 2.5 to 5 μg aliquots of total RNA was performed using random hexamers and Superscript III (Invitrogen). PCR was carried out on cDNA with gene specific primers. We verified the absence of contaminating genomic DNA in our RNA preparations by omitting the reverse transcriptase in the reverse transcription reactions and then performing PCR with exon-junction spanning primers to *β-actin *and *GAPDH*. We did not observe DNA contamination with this purification method.

### Array hybridization and microarray analysis

All RNA processing, array hybridizations, and scanning were performed by the Dana-Farber Cancer Institute MicroArray Core. Total RNA was processed using a 'Technology Access Gene Chip Eukaryotic Whole Transcript' protocol from Affymetrix (Santa Clara, CA, USA). Except for three modifications, this protocol is the same as that currently recommended for exon array processing in the [63]. We applied the following three modifications: 100 ng starting RNA (rather than 1 μg) was used; the rRNA reduction step was not carried out; and in the second cycle of cDNA synthesis, a second cDNA strand was synthesized before the clean-up and labeling steps. At the time these experiments and the array hybridizations were performed, the rRNA reduction step was not a part of the Affymetrix protocol. Biological triplicate RNA samples from untreated cells and cells KCl depolarized for 0.5, 1.5, 3, 6, 12, and 24 hours as well as cells exposed to TPG for 24 hours were analyzed on a total of 24 arrays. We quantile normalized all 24, then used the Affymetrix Probe Logarithmic Intensity Error algorithm to generate both exon and transcript level expression estimates. To select actual signal, we discarded from further data analysis those transcripts and exons belonging to the lower quartile of their respective datasets. These data have been deposited in National Center for Biotechnology Information's Gene Expression Omnibus (GEO) [64] and will be accessible through GEO series accession number GSE6976.

### Differential exon and transcript expression

Differential expression between each time point and the initial time point was tested via a moderated *t*-test, and an overall call for all times was generated via a moderated F-test [33]. Moderated *t*-tests and F-tests have the same interpretation as their ordinary equivalents. The difference is that standard errors have been moderated across probe sets by shrinking them toward a common value via an empirical Bayes procedure, which borrows power from the entire collection of probe sets. *P *values from the F-test were corrected for multiple testing to control the FDR, as described previously [34]. The FDR level for the transcript dataset was chosen to be 0.005. This value was empirically chosen from comparison with the receiver operating characteristic curve (Additional data file 6 [part B]). Because the exon probe sets contain fewer probes, and hence are subject to more noise, we selected a more relaxed FDR level of 0.01 for the exon dataset. For each selected probe set, a nested-F approach was applied to identify at what time points the probe set was significantly differentially expressed with respect to time 0. Each t-statistic was called significant if the F-test was still significant when all of the larger t-statistics were set to the same absolute size as the t-statistic in question. For each of the time points that passed the nested-F test, a fold change was computed. Only probe sets with a fold change of 1.5 or greater in at least one of the significant time points were retained in the analysis. The same procedure was used to test for differential expression between the TPG treatment and KCl treatment at 0 and 24 hours.

Because transcript estimates are a direct composite of individual exon use, we expected the two datasets to overlap significantly. Indeed, when an FDR of 1% was applied to the exon datasets, 72% (KCl) and 91% (TPG) of the components of the transcript datasets were recognized as containing significantly changing exons (Figure 2). The outlying fraction of the transcript dataset represents those transcripts for which no single exon passed the selection criteria. This occurs because of greater variability at the probe set level. Increasing the stringency of the exon FDR to 0.5% lowered the intersection to 52% (KCl) and 83% (TPG) of the transcript datasets. Although there is a drop off in the intersection, particularly between the KCl datasets, an 0.5% FDR exon cut-off still demonstrated similar trends between the transcript and exon datasets in the global response to elevated [Ca^2+^]_i_, as reflected in subsequent GO analyses (not shown).

### Clustering analysis

We applied a correlation analysis [35] to each list in order to separate both transcripts and exons into groups with similar expression profiles. Hierarchical clustering with a correlation-based distance was used to identify the six top classes of expression profiles. A representation of the typical trend in each class was obtained as follows. Each probe set expression profile was normalized by subtracting its mean and dividing by its standard deviation. All normalized expression values of all probe sets in the class in question were then pooled together and mean and standard deviation was computed at each time point:

$$\{\begin{array}{c} {\bar{\text{y}}}_{\text{j}}^{\text{C}}=\sum_{{\text{i}}_{\text{c}}\text{k}}{\bar{\text{y}}}_{{\text{i}}_{\text{c}}\text{jk}}/{\text{N}}_{\text{c}}{\text{n}}_{\text{r}}\\ \sigma_{\text{j}}^{\text{C}}=\sqrt{\sum_{{\text{i}}_{\text{c}}\text{k}}{\left({\bar{\text{y}}}_{{\text{i}}_{\text{c}}\text{jk}}-{\bar{\text{y}}}_{\text{j}}^{\text{C}}\right)}^{2}/\left({\text{N}}_{\text{C}}{\text{n}}_{\text{r}}-1\right)} \end{array}$$

Where the following are the normalized expression profiles:

$${\bar{\text{y}}}_{{\text{i}}_{\text{c}}\text{jk}}=\left({\text{y}}_{{\text{i}}_{\text{c}}\text{jk}}-\frac{1}{{\text{n}}_{\text{t}}{\text{n}}_{\text{r}}}\sum_{\text{jk}}{\text{y}}_{{\text{i}}_{\text{c}}\text{jk}}\right)/\sqrt{\frac{1}{{\text{n}}_{\text{t}}{\text{n}}_{\text{r}}-1}\sum_{\text{jk}}{\left({\text{y}}_{{\text{i}}_{\text{c}}\text{jk}}-\frac{1}{{\text{n}}_{\text{t}}{\text{n}}_{\text{r}}}\sum_{{\text{j}}^{\prime }{\text{k}}^{\prime }}{\text{y}}_{{\text{i}}_{\text{c}}{\text{j}}^{\prime }{\text{k}}^{\prime }}\right)}^{2}}$$

And where ${\text{y}}_{{\text{i}}_{\text{c}}\text{jk}}$ is the expression level for the k^th ^replicate at time j for gene i_C _in cluster C containing N_C _genes, and n_t _and n_r _are the total number of time points and replicates, respectively.

### PCR-based microarray validation

cDNA quantification was performed by semi-quantitative PCR and quantitative PCR. Primer sets targeting transcript regions were designed using Primers3 [65]. PCR was carried out in 40 μl reactions with Platinum Taq (Invitrogen). The linear amplification range for each primer pair/target amplicon was found empirically by titrating amounts of input cDNA. See Additional data file 6 for all oligonucleotides used in this study. PCR products were electrophoresed through either 8% polyacrylamide/Tris-Borate-EDTA buffer (TBE) or 2% to 4% agarose/Tris-Acetate-EDTA buffer (TAE) gels, stained with sybrGold (Invitrogen) or ethidium bromide, and visualized using a 595 FluorImager (ABI; Molecular Dynamics, Foster City, CA, USA) or an Alpha Imager (Alpha Innotech, San Leandro, CA, USA). In the case of quantitative PCR, signal intensities from TIF images were quantified using QuantityOne software (BioRad, Hercules, CA, USA).

### Gene Ontology

GO analyses were performed using the online tool GOstat [36,37]. To determine GO term enrichment, transcripts were compared against the complete set of annotated genes in the human GO gene associations database. The *P *value refers to the results of a Fisher's exact test. FDR (Benjamini) was used as the correction for multiple hypothesis testing.

### Cloning and DNA sequencing of splice forms

PCR products from splicing reactions were subcloned into the pCRII vector using the TOPO-TA Cloning Kit (Invitrogen), in accordance with the manufacturer's instructions. Subsequent colonies were selected for DNA inserts based on blue/white selection. DNA prepared from positive colonies was screened by EcoR1 digestion. Minipreps corresponding to unique restriction banding were submitted for automated sequencing to the Molecular Biology Core Facilities at the Dana-Farber Cancer Institute.

### Exon classification

Based on RefSeq gene annotations, exons of multi-exon genes were classified as constitutive, cassette, partially 5' spliced, or partially 3' spliced, according to the following criteria. Two exons 'agree' if there is one RefSeq isoform that contains them both. A constitutive exon is one that belongs to all isoforms. An exon was considered partially 5' spliced or 3' spliced if it shares the same chromosome, orientation, and either the 5' end-point or the 3' end-point with some other exon, respectively. If an exon is not partial 5' or partial 3', and not constitutive, then it is considered a cassette exon. Exons were also classified as to their position in a transcript. We evaluated an exon either as initial (5'), final (3'), or internal if it was the first, last, or positioned elsewhere, respectively, in a transcript. Note that this classification is not exclusive. An exon can be considered a starting exon of one isoform but an internal exon of another isoform. For each isoform position classification group, we counted the number of exons according to their splicing classification for all RefSeq isoforms and for each exon list. To assess statistical significance for enrichment, we took the overall genome exon splicing class proportions as parameters for a multinomial null distribution and used a binomial test on the counts for each class. The same procedure was used to compare exon dataset counts with GenBank mRNAs.

## Additional data files

The following additional data are available with the online version of this paper. Additional data file 1 is a figure of PCR data confirming the stimulus-induced transcription of known mRNAs. Additional data file 2 is a table containing the transcripts affected by elevated [Ca+]_i_. Additional data file 3 is a table containing the exons affected by elevated [Ca+]_i_. Additional data file 4 is a figure presenting the distribution of the number of exons identified as changing in the KCl datasets. Additional data file 5 is a table listing the GO categories enriched among transcripts affected by elevated [Ca+]_i_. Additional data file 6 is a figure presenting analyses of transcript and exon PCR validation. Additional data file 7 is a figure presenting quantification of transcript PCR validation. Additional data file 8 is a table listing the GO categories enriched among transcripts affected at the exon level by elevated [Ca+]_i _through KCl treatment. Additional data file 9 is a figure presenting examples of splice form diversity identified from array data and verified by PCR. Additional data file 10 is a table of PCR primers used in this study.

## Abbreviations

[Ca^2+^]_i_, intracellular Ca^2+^; ER, endoplasmic reticulum; FDR, false discovery rate; GO, gene ontology, NMD, non-sense mediated decay; PTC, premature stop codon; TPG, thapsigargin.

## Authors' contributions

AEM designed the study, evaluated the results, and wrote the paper. ASB designed the study, performed statistical analyses, evaluated the results, implemented the computational framework, supported the work, and wrote the paper. EAF designed the study and supported the work. PAS designed the study, supported the work, and wrote the paper. NN performed statistical analyses, evaluated the results, and implemented the computational framework. LEC performed statistical analyses, evaluated the results, and implemented the computational framework. CAM performed statistical analyses.

## Supplementary Material

Additional data file 1 Presented is a figure of PCR data confirming the stimulus-induced transcription of known mRNAs. Click here for file

Additional data file 2 Presented is a table containing the transcripts affected by elevated [Ca^2+^]_i_. Click here for file

Additional data file 3 Presented is a table containing the exons affected by elevated [Ca^2+^]_i_. Click here for file

Additional data file 4 Presented is a figure presenting the distribution of the number of exons called as changing in the KCl datasets. Click here for file

Additional data file 5 Presented is a table listing the GO categories enriched among transcripts affected by elevated [Ca^2+^]_i_. Click here for file

Additional data file 6 Presented is a figure showing analyses of transcript and exon PCR validation. Click here for file

Additional data file 7 Presented is a figure showing quantification of transcript PCR validation. Click here for file

Additional data file 8 Presented is a table listing the GO categories enriched among transcripts affected at the exon level by elevated [Ca^2+^]_i _through KCl treatment. Click here for file

Additional data file 9 Presented is a figure showing examples of splice form diversity identified from array data and verified by PCR. Click here for file

Additional data file 10 Presented is a table of PCR primers used in this study. Click here for file
