# Supplementary material for: Exon expression profiling reveals stimulus-mediated exon use in neural cells
Source: Genome Biol. 2007 Aug 2;8(8):R159. doi: 10.1186/gb-2007-8-8-r159 (PMC2374990; doi:10.1186/gb-2007-8-8-r159)
Supplement: Additional data file 1 — Presented is a figure of PCR data confirming the stimulus-induced transcription of known mRNAs. [file gb-2007-8-8-r159-S1.pdf]

Additional data file 1

A

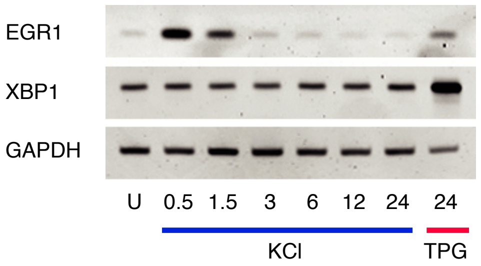

B

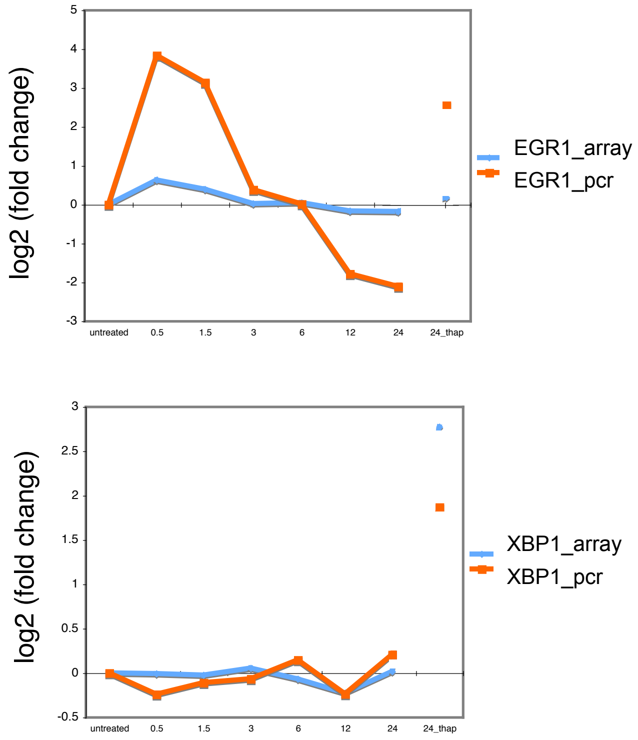

Validation of KCl and TPG-induced gene expression.

PCR data confirmed the stimulus-induced transcription of mRNAs known to be affected by KCl and TPG in similar neuroblastoma cells or in other neural systems. A) Human IMR-32 neuroblastoma cells were treated with 50 mM KCl for 0.5, 1.5, 3, 6, 12, or 24 hours or with 5 µM TPG for 24 hours. PCR performed on cDNA from untreated (U), KCl- or TPG-treated cells revealed temporal induction of the immediate early gene, early growth response (EGR1) in response to KCl and TPG as well as increased expression of the stress response X-box binding protein (XBP1) gene upon TPG treatment. Glyceraldehyde-3-phosphate dehydrogenase (GAPDH) served as a loading control. B) Quantification of the PCR products reveals a 14-fold up-regulation of EGR1 at the earliest KCl time point that returns to basal levels by 6 hrs. EGR1 is also induced upon prolonged TPG treatment. XBP1, in contrast, is selectively up-regulated by TPG. Shown are log (base 2) plots of the array and PCR data for these transcripts, normalized to GAPDH at each time point. Note that while the array data accurately determines the direction of change (either up- or down-regulated), the magnitude of change is often different than measured by qPCR.
