# Supplementary material for: Exon expression profiling reveals stimulus-mediated exon use in neural cells
Source: Genome Biol. 2007 Aug 2;8(8):R159. doi: 10.1186/gb-2007-8-8-r159 (PMC2374990; doi:10.1186/gb-2007-8-8-r159)
Supplement: Additional data file 4 — Presented is a figure presenting the distribution of the number of exons called as changing in the KCl datasets. [file gb-2007-8-8-r159-S4.pdf]

Additional data file 4

A

B

C

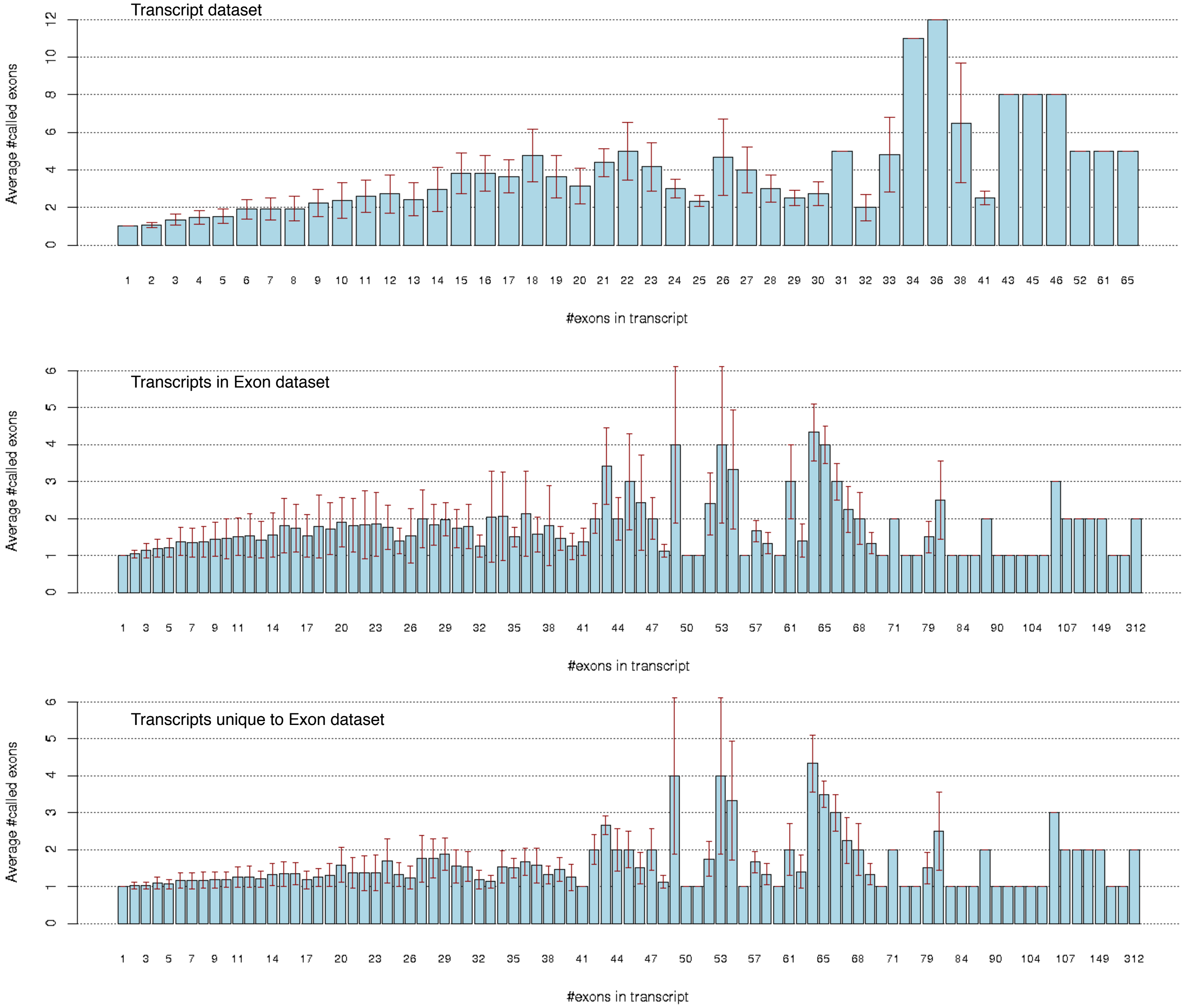

Distribution of changing exons per transcript under KCl treatment.

The average number of exons called as changing per transcript for transcripts of a given number of exons is shown. Note the different y-axis for the Transcript dataset. Greater numbers of exons per transcript were called as changing among those transcripts in the KCl-Transcript list. Error bars represent the standard deviation for all transcripts of the same exon number. Datasets used to generate each histogram were A) KCl-Transcript dataset, B) Transcripts of the KCl-Exon dataset (includes those transcripts in both the Transcript and Exon datasets), and C) Transcripts unique to the KCl-Exon dataset. Similar resulsts were obtained for the TPG datasets (data not shown).
