# Supplementary material for: Exon expression profiling reveals stimulus-mediated exon use in neural cells
Source: Genome Biol. 2007 Aug 2;8(8):R159. doi: 10.1186/gb-2007-8-8-r159 (PMC2374990; doi:10.1186/gb-2007-8-8-r159)
Supplement: Additional data file 5 — Presented is a table listing the GO categories enriched among transcripts affected by elevated [Ca2+]i. [file gb-2007-8-8-r159-S5.pdf]

Additional data file 5

Gene Ontology Categories Enriched in the KCl-Transcript dataset

Gene Ontology Category	Transcript Cluster 1 down-regulated	p-value	Transcript Cluster 2 up-regulated	p-value	Transcript Cluster 3 TPG-up-regulated	p-value	Transcript Cluster 4 intermediate-up-regulated	p-value	total
nucleotide metabolism	81	6.55E-20							1227
lipid metabolism	45	1.16E-21							468
glucose metabolism	22	1.67E-11							214
cell cycle	82	9.28E-30							871
mitochondria	100	3.65E-58							771
cellular metabolism	456	1.16E-26							12279
intrinsic plasma membrane			34	1.14E-19					1363
ion transport			20	3.53E-03					1528
cation channel activity			10	5.56E-03					443
calcium ion binding			20	2.16E-03					1348
nucleosome					7	1.89E-06			122
ER-Golgi intermediate compartment					3	1.08E-03			31
Retrograde vesicle trafficking					2	7.03E-03			16
transcription from Pol II promoters							3	1.44E-03	429

Number of transcripts and the p-value associated with each gene ontology category per Transcript Cluster. The final column lists the total number of genes in each GO category.
