# Supplementary material for: Exon expression profiling reveals stimulus-mediated exon use in neural cells
Source: Genome Biol. 2007 Aug 2;8(8):R159. doi: 10.1186/gb-2007-8-8-r159 (PMC2374990; doi:10.1186/gb-2007-8-8-r159)
Supplement: Additional data file 6 — Presented is a figure showing analyses of transcript and exon PCR validation. [file gb-2007-8-8-r159-S6.pdf]

Additional data file 6

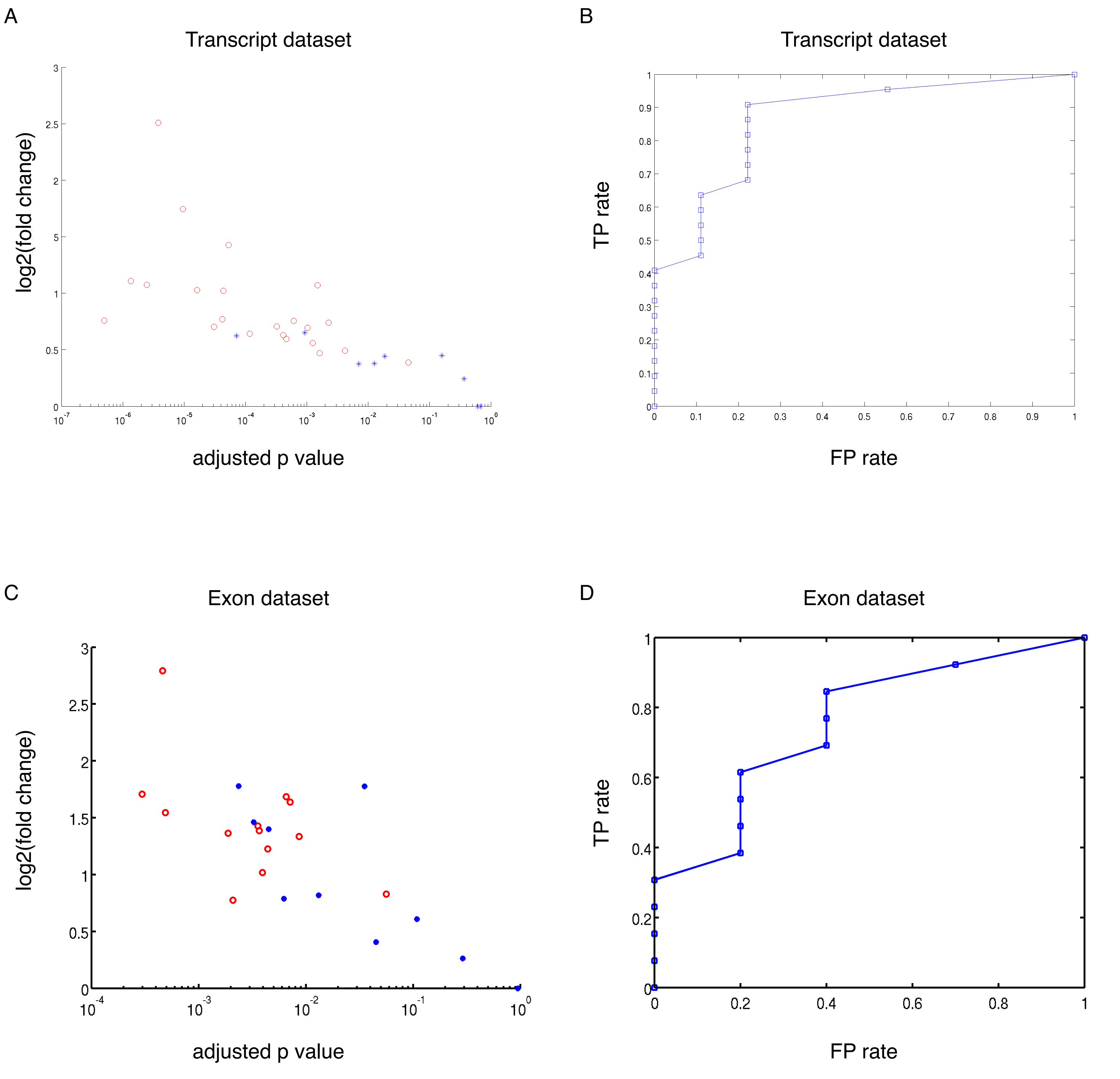

Validation of Microarray Transcript-Level and Exon-Level Data by qPCR.

A, C) Maximum log-fold-change (over time) versus adjusted p-value of the genes tested with PCR obtained from the statistical analysis of the Transcript and Exon datasets, respectively. Red circles correspond to genes with a significant expression change over time according to qPCR, blue stars correspond to genes with no significant expression change over time. The absolute value of the fold change called by the array is plotted on the y-axis. B, D) Receiver Operating Characteristic (ROC) curve obtained from the genes tested with PCR by varying the adjusted p-value threshold in the statistical analysis of the Transcript or Exon dataset for a fixed fold-change threshold of 1.5. The y-axis reports the True Positive rate (TP rate) as a function of the False Positive rate (FP rate, x-axis).
