# Supplementary material for: Exon expression profiling reveals stimulus-mediated exon use in neural cells
Source: Genome Biol. 2007 Aug 2;8(8):R159. doi: 10.1186/gb-2007-8-8-r159 (PMC2374990; doi:10.1186/gb-2007-8-8-r159)
Supplement: Additional data file 7 — Presented is a figure showing quantification of transcript PCR validation. [file gb-2007-8-8-r159-S7.pdf]

Additional data file 7

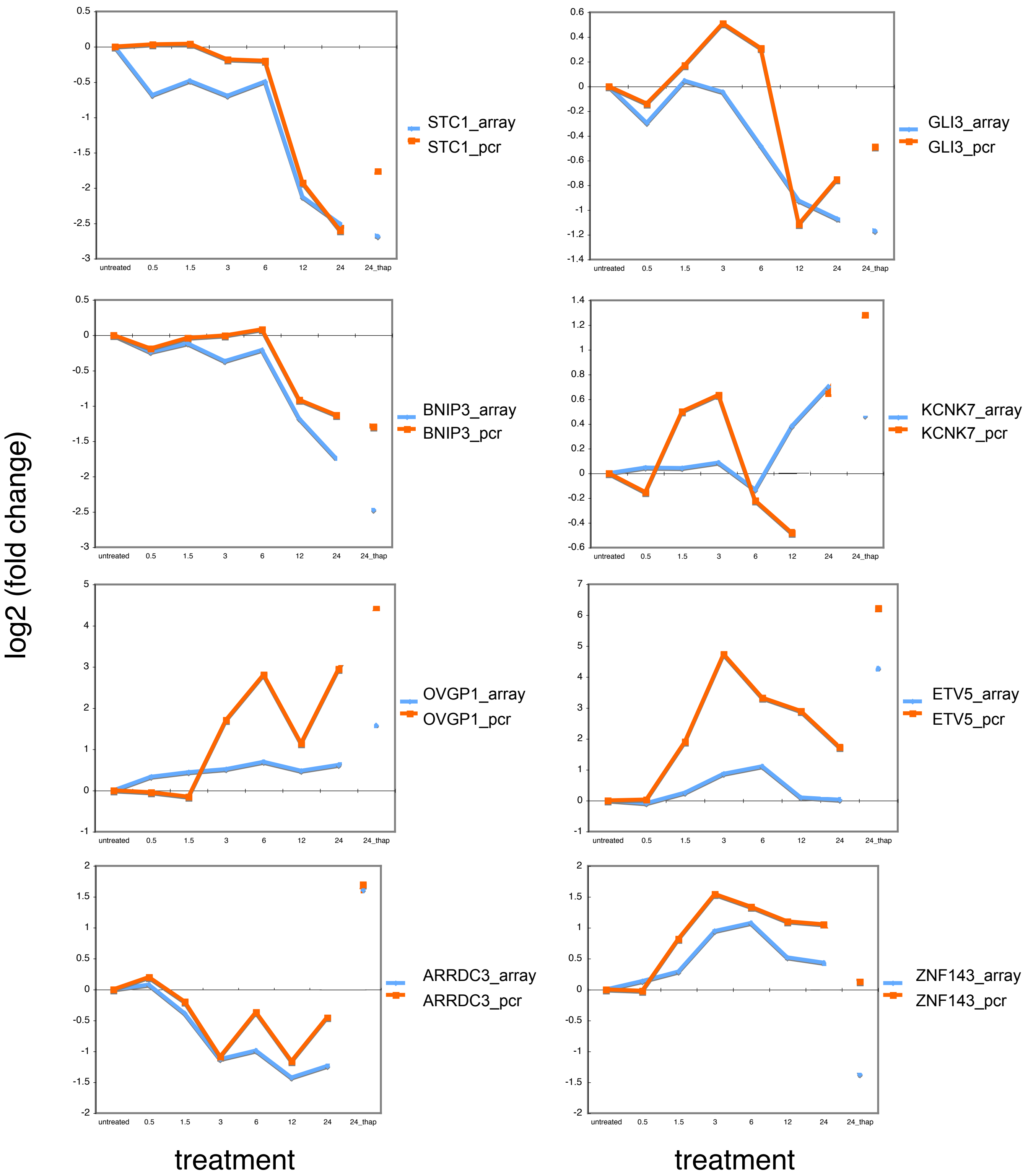

Quantification of Transcript PCR Validation.

For each transcript, the log (base 2) of the fold change as determined by the array and by quantification of PCR data are plotted by time point. Note that while the array data accurately determines the direction of change (either up- or down-regulated), the magnitude of change is often different than measured by PCR. qPCR values at each time point were normalized to the amount of PREX1, a gene not predicted to change in abundance. Gene names are listed in Figure 3.
