# Supplementary material for: Exon expression profiling reveals stimulus-mediated exon use in neural cells
Source: Genome Biol. 2007 Aug 2;8(8):R159. doi: 10.1186/gb-2007-8-8-r159 (PMC2374990; doi:10.1186/gb-2007-8-8-r159)
Supplement: Additional data file 8 — Presented is a table listing the GO categories enriched among transcripts affected at the exon level by elevated [Ca2+]i through KCl treatment. [file gb-2007-8-8-r159-S8.pdf]

Additional data file 8

Gene Ontology Categories Enriched in the KCl-Exon dataset

Gene Ontology Category	0.5h	p-value	1.5h	p-value	3h	p-value	6h	p-value	12h	p-value	24h	p-value	total
apoptosis									118	5.91E-12	122	1.28E-12	692
calcium ion binding	53	0.00344	51	4.07E-05	46	0.000908	70	4.02E-05	176	3.09E-06	178	8.03E-06	1348
calmodulin binding	13	0.000975	12	0.000813					39	6.58E-13	36	1.17E-09	139
cell adhesion	62	3.47E-15	44	1.23E-07	54	3.11E-16	51	0.000325	137	1.01E-07	142	2.38E-08	946
cell cycle							55	3.72E-07	178	2.49E-30	195	3.07E-39	871
cellular metabolism							445	5.40E-07	1375	1.45E-25	1434	1.94E-30	12279
endoplasmic recticulum									129	1.70E-17	132	8.02E-18	685
intrinsic plasma membrane	94	5.30E-26	70	6.58E-16	71	8.97E-18	65	0.0017	204	3.07E-13	203	1.34E-11	1363
mitochondria									155	3.70E-25	151	2.17E-21	771
mRNA processing									64	4.23E-17	68	1.47E-21	243
RNA splicing									39	1.96E-11	43	1.75E-14	148
transcription							37	7.88E-07	100	2.67E-15	108	6.65E-19	507
phosphate transport	22	2.64E-11	16	0.000274	14	0.00195							215
regulation of kinase activity	16	2.80E-05			10	0.00996							149
protein kinase cascade							28	2.35E-08					303

Number of genes and the p-value associated with each gene ontology category, by time point.
The final column lists the total number of genes in each GO category.
