# Supplementary material for: Exon expression profiling reveals stimulus-mediated exon use in neural cells
Source: Genome Biol. 2007 Aug 2;8(8):R159. doi: 10.1186/gb-2007-8-8-r159 (PMC2374990; doi:10.1186/gb-2007-8-8-r159)
Supplement: Additional data file 9 — Presented is a figure showing examples of splice form diversity identified from array data and verified by PCR. [file gb-2007-8-8-r159-S9.pdf]

Additional data file 9

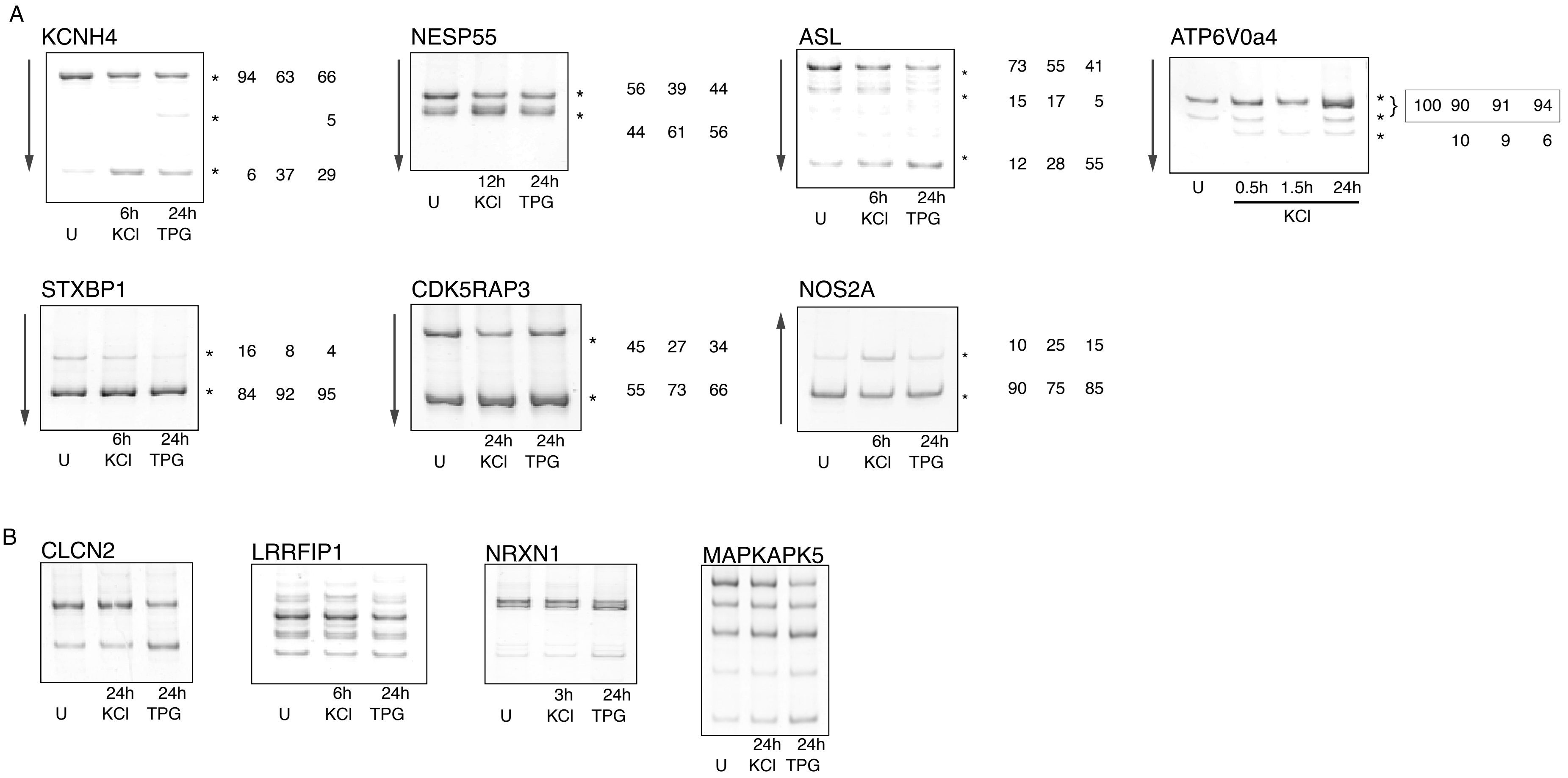

Examples of Splice Form Diversity Identified from Exon Array Data.

A) Microarray data detected changes in the expression of specific exons within KCNH4, NESP55, ASL, ATP6V0a4, STXBP1, CDK5RAP3, and NOS2A. For each gene, PCR was performed on cDNA from KCl or TPG treated cells using primers to amplify across the exon of interest. Multiple splice froms were detected that indicated regulated alternative splicing in the vicinity of the identified exons, as predicted. Arrows indicate the behavior of the exon, as measured by the array. Quantification of splice forms is presented as a percentage of the bands identifed (*). B) Multiple isoforms were dected for four additional transcripts but with ambigusous (LRRFIP1, NRXN1, MAPKAPK5) or opposite (CLCN2) regulation as predicted by the array. The KCl time point shown is indicated. CLCN2, chloride channel 2, LRRFIP1, leucine rich repeat (in FLII) interacting protein 1, NRXN1, neurexin 1, MAPKAPK5, mitogen-activated protein kinase-activated protein kinase 5. Other gene names are listed in the text and in Figure 4.
